# m^6^A methylation: a potential key player in understanding and treating COVID-2019 infection

**DOI:** 10.1038/s41420-023-01580-1

**Published:** 2023-08-18

**Authors:** Weiwei Qian, Jian Zhou, Ligeng Duan, Haoyu Wang, Shuyun Xu, Yu Cao

**Affiliations:** 1 Department of Emergency Medicine, Laboratory of Emergency Medicine, West China Hospital, and Disaster Medical Center, Sichuan University, Chengdu, 610044 Sichuan China; 2 Department of Emergency,Shangjinnanfu Hospital, West China Hospital, Sichuan University, Chengdu, 610044 Sichuan China; 3 Department of Immunology, International Cancer Center, Shenzhen University Health Science Center, Shenzhen, 518061 Guangdong China; 4 Guangdong Key Laboratory for Biomedical Measurements and Ultrasound Imaging, National-Regional Key Technology Engineering Laboratory for Medical Ultrasound, School of Biomedical Engineering, Shenzhen University Medical School, Shenzhen, 518060 China

**Keywords:** Infectious diseases, Immunology

## Abstract

Since its discovery in 2019, coronavirus disease 2019 (COVID-2019) spans a wide clinical spectrum from the asymptomatic stage, mild infection, to severe pneumonia. In patients with COVID-2019, factors such as advanced age, diabetes, or hypertension are associated with a significantly increased risk of severe diseases and death. Of note, the mechanisms underlying differences in the risk and symptoms of COVID-2019 among different populations are still poorly characterized. Accordingly, it is imperative to elucidate potential pathophysiological mechanisms and develop targeted therapeutic approaches for COVID-2019 infection. N6-methyladenosine (m^6^A) is one of the most common modifications in mammalian RNA transcripts and is widely found in messenger RNAs and some non-coding RNAs. It has been reported that m^6^A methylation modifications are present in viral RNA transcripts, which are of great significance for the regulation of the viral life cycle. Furthermore, m^6^A methylation has recently been found to be strongly associated with COVID-2019 infection. Therefore, this article reviews recent advances in studies related to the role of m^6^A methylation in COVID-2019 infection.

## Facts

1. This article systematically reviews research on m6A and COVID-2019 infection;
2. This article proposes potential future research directions on the relationship between m6A and COVID-2019 infection in the context of existing research.

## Open Questions

1. This article suggests a mathematical model based on m6A-related genes for the prediction of COVID-2019 infection;
2. This article proposes a treatment strategy for COVID-2019 infection based on m6A-related pathways.

## Introduction

The novel coronavirus is an RNA virus that belongs to the family Coronaviridae and is a close relative of the severe acute respiratory syndrome (SARS) and the Middle East respiratory syndrome (MERS), whose full name is Severe Acute Respiratory Syndrome Coronavirus 2 (SARS-CoV-2) [1]. This virus is the pathogen causing coronavirus disease 2019 (COVID-19) [2]. COVID-19 is a highly infectious and lethal disease that is majorly transmitted between individuals through respiratory droplets and contact routes and has caused hundreds of millions of infections and millions of deaths worldwide, for which there is no specific treatment [3–7].Some drugs, such as remdesivir, chloroquine, and hydroxychloroquine, have been used in the treatment of COVID-19 [8], but their efficacy and safety still require further research. Currently, COVID-19 patients are mainly treated with supportive care, including oxygen therapy, fluid infusion, and organ support, which aims to enable patients to recover as well as possible [9]. It has become a hot research topic to investigate the pathogenesis of COVID-19 at the molecular level and identify reliable therapeutic targets.

N6-methyladenosine (m^6^A) is a common RNA modification and widely exists in cells [10]. m^6^A involves the addition of methyl groups to RNA molecules and plays an important role in biological processes such as transcription, translation, and splicing of RNA [11–13] . Recently, mounting evidence has revealed that m^6^A methylation modifications are closely related to virus infection [14–19]. As many studies demonstrated, m^6^A methylation can affect cellular function through various mechanisms during the occurrence of hepatitis virus, human immunodeficiency virus type 1, and SARS-CoV-2 infection to adapt to the life cycle demands of the virus [15, 16, 18, 20–23]. In turn, m^6^A methylation can also have an impact on viral infection, affecting the life cycle and pathogenicity of the virus [20, 24, 25] Therefore, elucidating the specific mechanism of m^6^A methylation in COVID-19 virus infection is important for the prevention and treatment of COVID-19.

## RNA m^6^A methylation

### Overview of m^6^A

RNA m^6^A methylation refers to a methylation modification that occurs at the nitrogen-6 position of the adenine (A) base on RNA molecules, which is widely found in eukaryotic ribosomal messenger RNA (mRNA), transfer RNA (tRNA), RNA (rRNA), and noncoding RNA (ncRNA) and has been shown to be important in regulating various biological processes of RNA [10, 26, 27]. As one of the most common RNA modifications, m^6^A methylation occurs mainly in the shared sequence RRACH (R = G or A; H = A, C, or U) and is mostly enriched in the stop codon, 3' untranslated region, and long internal exons, which is dynamically reversible under the regulation of various modifying enzymes, such as methyltransferases (writers), demethylases (erasers), and methylated readers (readers) [28]. m^6^A methylation is catalyzed by methyltransferases. Furthermore, two methyltransferases have been identified to be involved in m^6^A methylation, including methyltransferase-like 3 (METTL3) and METTL14 [29]. In addition, other proteins, such as WTAP, KIAA1429, and RBM15, are also implicated in the mediation of m^6^A methylation [30]. m^6^A readers are a class of proteins that recognize and bind to m^6^A-modified RNAs, which include many proteins, such as YTHDC1, YTHDC2, YTHDF1, YTHDF2, YTHDF3, HNRNPC, and IGF2BP1 [31]. These proteins can regulate biological processes of RNA, including transcription, splicing, translation, and degradation [31–33]. Demethylases can reverse the m^6^A methylation of RNA by reducing the m^6^A modification to A. A variety of demethylases have been identified, including FTO and ALKBH5, which are involved in RNA metabolism and regulation and are essential for maintaining RNA stability and function [33]. m^6^A recognition factors are a class of RNA structural domains capable of recognizing and binding to m^6^A modifications, which consist of YTHDC1, YTHDC2, YTHDF1, YTHDF2, and YTHDF3. Moreover, m^6^A recognition factors participate in the recognition and interaction to RNA and modulate the local structure and interaction of RNA [34].

## m^6^A and COVID-2019 infection

### Effects of m^6^A methylation on COVID-2019 infection

**Fig. 1:**
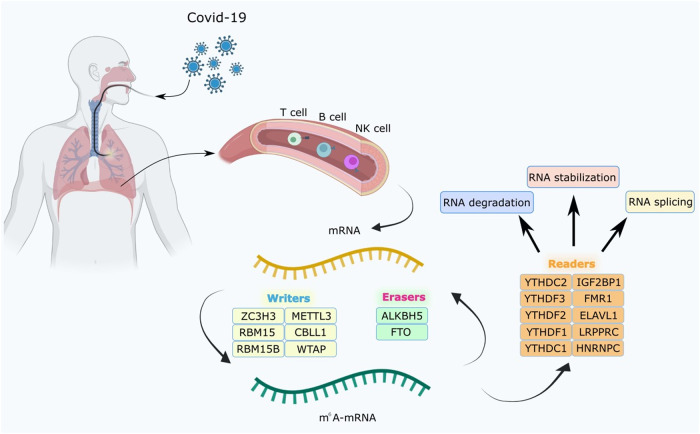
The overview of m^6^A RNA methylation modification in blood lymphocytes of patients infected with SARSCoV-2, including writers, readers, and erasers.

Accumulating evidence has unraveled that m^6^A modification is crucial for the transmission and pathogenicity of COVID-2019. For instance, Liu et al. found that m^6^A modification was deregulated in host cells infected with SARS-CoV-2 and re-localized by viral infection, thereby increasing the abundance of m^6^A in cells [17]. Campos et al. identified the methylation of m^6^A transcripts in African green monkey Vero cells and human Calu-6 cells infected with SARS-CoV-2 with RNA sequencing and analyzed the results with nonparametric statistics and two computational methods (m6anet and EpiNano), which showed higher levels of m^6^A methylation in the RNA of infected cells [35]. This study verifies that the effect of SARS-CoV-2 virus infection on cell function is associated with m^6^A methylation. Qiu et al. observed downregulation of m^6^A-related genes in blood leukocytes from COVID-2019 patients [36]. In addition, a study by Li et al. showed that the m^6^A methylation gene METTL3 was significantly downregulated and inflammatory genes were upregulated in lung tissues of patients with severe COVID-2019 than in those of healthy individuals [16]. Additionally, Lu et al. analyzed the expression of 9 writers, 15 readers, and 2 erasers for m^6^A modification in healthy individuals and COVID-2019 patients and observed that the expression of WTAP, RBM15, HNRNPC, YTHDC1, FMR1, HNRNPA2B1, ELAVL1, and YTHDF3 was markedly higher but the expression of RBM15B, IGFBP2, and IGF2BP1 was substantially poorer in COVID-2019 patients than in healthy individuals, illustrating that the above targets may be associated with the regulation of m^6^A on COVID-2019 infection [37] (Fig. 1). Subsequent research further validated that SARS-CoV-2 infection altered the epigenetic transcriptome of m^6^A in lymphocytes, especially in those from patients with severe disease, and that it could enhance RBM15 m^6^A modification to regulate host immune responses [38].

The pathogenesis of severe respiratory infections caused by novel coronaviruses is associated with a disorder of the pro-inflammatory/anti-inflammatory response and immune imbalance, with immune system overreaction, excessive apoptosis and depletion of immune cells, immunosuppression, and immunoparalysis as the main immunopathological manifestations. For instance, prior research revealed that patients with severe COVID-2019 had high levels of pro-inflammatory cytokines than patients with moderate COVID-2019, indicating that high levels of pro-inflammatory cytokines are associated with the poor prognosis of COVID-2019 patients [39, 40]. Braun et al. found peak reactivity of CD35 T cells in 4% of COVID-2019 patients and 19% of healthy controls, illustrating the existence of T cell cross-reactivity which may prevent SARS-CoV-2 infection to some extent [41]. Because persistent inflammatory factor storms serve as one of the most dangerous factors in the COVID-19 pandemic, pro-inflammatory cytokines such as IL-1α, IL-1β, IL-6, and TNF-α play a key role in COVID-19 development. Recent studies have indicated that m^6^A modification can affect SARS-CoV-2 infection by regulating the antiviral response of immune cells. A previous study exhibited that METTL3 was downregulated in host cells after SARS-CoV-2 infection to decrease m^6^A levels in viral and host genes, thus enhancing retinoic acid-inducible gene-I binding and the expression of downstream innate immune pathways and inflammatory genes, suggesting that human immune responses play an important role in COVID-2019 infection [42]. A study by Du et al. unraveled that deletion of the m^6^A-related target METTL14 or YTHDF1 in macrophages predisposed infected mice to inflammatory factor storms, resulting in higher mortality from infection, which illustrated m^6^A as a pivotal mechanism regulating inflammatory factor storms [43]. In summary, the immune response participates in the development and progression of COVID-2019. As an important biomolecule, m^6^A not only is essential for gene expression, translation, and regulation but also exerts important physiological functions in various aspects of immune responses in the body. The role of m^6^A in the immune system has been investigated, which provides not only a novel perspective for the in-depth understanding of the biological function of m^6^A but also a critical reference for exploring the occurrence, transmission, prevention, and control of COVID-2019 [44–46]. However, there is a lack of research on the association of m^6^A with immune responses in COVID-2019 infections. In this context, further research is warranted to characterize the regulatory mechanism of m^6^A in immune cells and the potential molecular mechanism of interactions between m^6^A and COVID-2019 infection-related immune responses, thus providing new ideas and approaches for the treatment and prevention of COVID-2019.

### Effect of m^6^A methylation modification on the evolution of SARS-CoV-2

It has been demonstrated that m^6^A methylation modifications also play a crucial role in the transmission and evolution of SARS-CoV-2. Liu et al. used RNA immunoprecipitation sequencing for sequencing of the m^6^A methylation profiles in SARS-CoV-2-infected human and monkey cells and systematic bioinformatics analysis and found the dynamic distribution pattern of m^6^A modifications in the SARS-CoV-2 genome and that m^6^A modifications were also widely present on the negative-strand RNAs as replication intermediates. Further, the researchers used the more precise m^6^A individual-nucleotide-resolution cross-linking and immunoprecipitation technique to identify m^6^A sites at single-base resolution on SARS-CoV-2 genomic RNA and identified eight m^6^A sites at single-base resolution, and more importantly, phylogenetic analysis unveiled that a series of mutant strains carrying specific m^6^A modification site emerged in different regions of the world. Functional experiments confirmed that the m^6^A methyltransferase METTL3/14 and the demethylase ALKBH5 negatively/positively regulated the replication of the SARS-CoV-2 genome, respectively, and that a decrease in the m^6^A reading protein YTHDF2 also promoted viral replication and infectivity. As this result indicated, SARS-CoV-2 infection not only elevates m^6^A contents in the host genome and changes the overall methylation profile. Meanwhile, SARS-CoV-2 can also use cellular enzymes for its own methylation to generate an evolutionary pressure to adapt the viral DRACH sequence, thus becoming more similar to the cellular sequence to successfully evade the killing effect of interferon. In conclusion, m^6^A modifications affect the interaction between the host and SARS-CoV-2 [17]. The research by Wyler et al. also revealed that m^6^A methylation is vital for virus-host interactions [47]. It was found in the research by Zhang et al. that METTL3A expression was increased and its distribution in cells was changed after SARS-CoV-2 infection. This research also confirmed the effect of METTL3 on viral replication by knocking down or overexpressing METTL3 through transfection with short hairpin RNA or plasmids [25].

## m^6^A methylation modifications and diagnosis of SARS-CoV-2 infection

The importance of m^6^A in COVID-2019 infection was previously confirmed. Accordingly, molecular mechanisms underlying m^6^A can be used for the development of new preventive and therapeutic options for COVID-2019. In the study by Lu et al., a model to predict the risk of COVID-2019 was constructed by screening m^6^A-related genes, which successfully predicted the degree of risk of COVID-2019 infection and had a high validity [48]. In the study by Qing et al., 18 basic m^6^A methylation enzymes were identified, among which the eight optimal m^6^A methylation enzymes were selected to predict the occurrence of COVID-2019 with a random forest model which had high-accuracy prediction capability [41]. As a result, the risk of COVID-2019 infection can be assessed by constructing a linear model of m^6^A-related genes. In addition, Dong et al. used LASSO and multivariate Cox regression analyses to determine prognostic factors for patients with COVID-2019 and found that hypertension, increased neutrophil-to-lymphocyte ratio, and elevated NT-proBNP were significantly associated with the poor in-hospital prognosis of COVID-2019 patients. The model involving these three factors effectively predicted the in-hospital survival of COVID-2019 patients, which, therefore, is expected to be applied to the clinical management of COVID-2019 [49]. Therefore, the prediction models of m^6^A-related genes constructed based on clinical symptoms would be expected to more accurately predict disease onset and progression in the early stages of COVID-2019 infection.

## m^6^A methylation modification and treatment of SARS-CoV-2 infection

As reported, m^6^A-related regulators in SARS-CoV-2 infection provide new strategies for the development of vaccines and antiviral drugs. Specifically, m^6^A modification-related genes are knocked down or overexpressed to reduce viral virulence, thus designing attenuated vaccine viruses. Additionally, the mechanisms of m^6^A modification can be utilized as a new target for antiviral therapy, such as the method investigated by Aik et al. [50]. Malacrida et al. [51] reported a variety of m^6^A demethylation-related small molecules, such as N-oxalylglycine (NOG), 2,4-Pyridinedicarboxylic acid (2,4-PDCA), IOX3, and MV1035, providing ideas for the development of drugs involving m^6^A-related targets. Liu et al. found that knockdown of METTL3, METTL14, and YTHDF2 increased viral replication, whereas knockdown of ALKBH5 repressed SARS-CoV-2 infection [17]. Therefore, drugs targeting these relevant targets can be more effective in treating COVID-2019 infection. A study by Zannella et al. exhibited that emodin enhanced interference with the replication of β-coronavirus replication, particularly SARS-CoV-2, and that its strong activity of anti-SARS-CoV-2 was associated with m^6^A pathways [52]. There are some reports on the antiviral effects of m^6^A-related small molecule drugs (such as NOG, 2,4-PDCA, IOX3, and imidazobenzoxazin-5-thione MV1035) against other viruses. Nevertheless, the development of m^6^A-related small molecule drugs against SARS-CoV-2 requires more in-depth research, which helps to increase the efficacy of treatment of COVID-2019 infection and provides novel ideas and methods to prevent similar outbreaks.

## Conclusion and outlook

With the global outbreak of the COVID-2019 epidemic, tremendous attention has been paid to the association between m^6^A methylation and COVID-2019 infection, and the involved mechanisms are being intensively explored. Although m^6^A methylation has been revealed to play a critical role in COVID-2019 infection, the detailed regulatory mechanisms still need in-depth studies. In addition, the development of m^6^A-related small molecule drugs against SARS-CoV-2 is also an important field, which needs to be intensively researched for more rapid and effective treatment of COVID-2019 infection to make a greater contribution to global human health security. In addition to the development of drugs targeting m^6^A methylation, research on other m^6^A-related molecular targets, such as reader proteins and demethylases, are also promising targets for vaccines and therapies against COVID-2019. In addition, as the understanding of m^6^A methylation gradually deepens, new therapeutic strategies may emerge, such as modulation of the immune response or suppression of inflammation by regulating m^6^A methylation modifications. Therefore, more vaccines and treatments targeting m^6^A methylation against COVID-2019 are expected to be developed in the future, providing new strategies and methods to control COVID-2019 outbreaks.

## Data Availability

The data that support the findings of this study are available from the corresponding author upon reasonable request.

## References

[1] Coronaviridae Study Group of the International Committee on Taxonomy of Viruses. (2020). The species Severe acute respiratory syndrome-related coronavirus: classifying 2019-nCoV and naming it SARS-CoV-2. Nat Microbiol.

[2] Hu B, Guo H, Zhou P, Shi ZL (2021). Characteristics of SARS-CoV-2 and COVID-19. Nat Rev Microbiol.

[3] Andreas M, Piechotta V, Skoetz N, Grummich K, Becker M, Joos L (2021). Interventions for palliative symptom control in COVID-19 patients. Cochrane Database Syst Rev.

[4] Ko HK, Yu WK, Pan SW, Chen WC, Yang KY, Lin YT (2022). Consensus statement and recommendations on the treatment of COVID-19: 2021 update. J Chin Med Assoc.

[5] Long B, Carius BM, Chavez S, Liang SY, Brady WJ, Koyfman A (2022). Clinical update on COVID-19 for the emergency clinician: Presentation and evaluation. Am J Emerg Med.

[6] Harrison AG, Lin T, Wang P (2020). Mechanisms of SARS-CoV-2 transmission and pathogenesis. Trends in immunology.

[7] Rehman SU, Rehman SU, Yoo HH (2021). COVID-19 challenges and its therapeutics. Biomed Pharmacothe.

[8] Szendrey M, Guo J, Li W, Yang T, Zhang S (2021). COVID-19 drugs chloroquine and hydroxychloroquine, but not azithromycin and remdesivir, block hERG potassium channels. J Pharmacol Exp Ther.

[9] Yuan Y, Jiao B, Qu L, Yang D, Liu R (2023). The development of COVID-19 treatment. Front Immunol.

[10] Oerum S, Meynier V, Catala M, Tisné C (2021). A comprehensive review of m6A/m6Am RNA methyltransferase structures. Nucleic Acids Res.

[11] Deng LJ, Deng WQ, Fan SR, Chen MF, Qi M, Lyu WY (2022). m6A modification: recent advances, anticancer targeted drug discovery and beyond. Mol Cancer.

[12] Tang Y, Chen K, Song B, Ma J, Wu X, Xu Q (2021). m6A-Atlas: a comprehensive knowledgebase for unraveling the N6-methyladenosine (m6A) epitranscriptome. Nucleic Acids Res.

[13] Wang X, Lu Z, Gomez A, Hon GC, Yue Y, Han D (2014). N6-methyladenosine-dependent regulation of messenger RNA stability. Nature.

[14] Chelmicki T, Roger E, Teissandier A, Dura M, Bonneville L, Rucli S (2021). m(6)A RNA methylation regulates the fate of endogenous retroviruses. Nature.

[15] Kostyusheva A, Brezgin S, Glebe D, Kostyushev D, Chulanov V (2021). Host-cell interactions in HBV infection and pathogenesis: the emerging role of m6A modification. Emerg Microbes Infect.

[16] Li N, Hui H, Bray B, Gonzalez GM, Zeller M, Anderson KG (2021). METTL3 regulates viral m6A RNA modification and host cell innate immune responses during SARS-CoV-2 infection. Cell Rep.

[17] Liu J, Xu YP, Li K, Ye Q, Zhou HY, Sun H (2021). The m(6)A methylome of SARS-CoV-2 in host cells. Cell Res.

[18] Yue J, Wei Y, Zhao M (2022). The reversible methylation of m6A is involved in plant virus infection. Biology (Basel).

[19] Zhang C, Dai D, Zhang W, Yang W, Guo Y, Wei Q (2022). Role of m6A RNA methylation in the development of hepatitis B virus-associated hepatocellular carcinoma. J Gastroenterol Hepatol.

[20] Brocard M, Ruggieri A, Locker N (2017). m6A RNA methylation, a new hallmark in virus-host interactions. J Gen Virol.

[21] Liao L, He Y, Li SJ, Zhang GG, Yu W, Yang J (2022). Anti-HIV drug elvitegravir suppresses cancer metastasis via increased proteasomal degradation of m6A methyltransferase METTL3. Cancer research.

[22] Wang JN, Wang F, Ke J, Li Z, Xu CH, Yang Q (2022). Inhibition of METTL3 attenuates renal injury and inflammation by alleviating TAB3 m6A modifications via IGF2BP2-dependent mechanisms. Sci Transl Med.

[23] Pereira-Montecinos C, Toro-Ascuy D, Ananías-Sáez C, Gaete-Argel A, Rojas-Fuentes C, Riquelme-Barrios S (2022). Epitranscriptomic regulation of HIV-1 full-length RNA packaging. Nucleic acids Res.

[24] Burgess HM, Depledge DP, Thompson L, Srinivas KP, Grande RC, Vink EI (2021). Targeting the m(6)A RNA modification pathway blocks SARS-CoV-2 and HCoV-OC43 replication. Genes Dev.

[25] Zhang X, Hao H, Ma L, Zhang Y, Hu X, Chen Z (2021). Methyltransferase-like 3 modulates severe acute respiratory syndrome Coronavirus-2 RNA N6-methyladenosine modification and replication. mBio.

[26] Dominissini D, Moshitch-Moshkovitz S, Schwartz S, Salmon-Divon M, Ungar L, Osenberg S (2012). Topology of the human and mouse m6A RNA methylomes revealed by m6A-seq. Nature.

[27] Zhang H, Shi X, Huang T, Zhao X, Chen W, Gu N (2020). Dynamic landscape and evolution of m6A methylation in human. Nucleic Acids Res.

[28] Schwartz S, Mumbach MR, Jovanovic M, Wang T, Maciag K, Bushkin GG (2014). Perturbation of m6A writers reveals two distinct classes of mRNA methylation at internal and 5’ sites. Cell Rep.

[29] Liu P, Li F, Lin J, Fukumoto T, Nacarelli T, Hao X (2021). m(6)A-independent genome-wide METTL3 and METTL14 redistribution drives the senescence-associated secretory phenotype. Nat Cell Biol.

[30] Jiang X, Liu B, Nie Z, Duan L, Xiong Q, Jin Z (2021). The role of m6A modification in the biological functions and diseases. Signal Trans Targeted Therapy.

[31] Zaccara S, Ries RJ, Jaffrey SR (2019). Reading, writing and erasing mRNA methylation. Nat Rev Mol Cell Biol.

[32] Li J, Ahn JH, Wang GG (2019). Understanding histone H3 lysine 36 methylation and its deregulation in disease. Cell Mol Life Sci.

[33] Fu Y, Dominissini D, Rechavi G, He C (2014). Gene expression regulation mediated through reversible m^6^A RNA methylation. Nat Rev Genet.

[34] Shi H, Wei J, He C (2019). Where, when, and how: context-dependent functions of RNA methylation writers, readers, and erasers. Mol Cell.

[35] Campos JHC, Alves GV, Maricato JT, Braconi CT, Antoneli FM, Janini LMR (2022). The epitranscriptome of Vero cells infected with SARS-CoV-2 assessed by direct RNA sequencing reveals m6A pattern changes and DRACH motif biases in viral and cellular RNAs. Front Cell Infect Microbiol.

[36] Qiu X, Hua X, Li Q, Zhou Q, Chen J (2021). m(6)A regulator-mediated methylation modification patterns and characteristics of immunity in blood leukocytes of COVID-19 patients. Front Immunol.

[37] Lu L, Li Y, Ao X, Huang J, Liu B, Wu L (2022). The risk of COVID-19 can be predicted by a nomogram based on m6A-related genes. Infect Genet Evol.

[38] Meng Y, Zhang Q, Wang K, Zhang X, Yang R, Bi K (2021). RBM15-mediated N6-methyladenosine modification affects COVID-19 severity by regulating the expression of multitarget genes. Cell Death Dis.

[39] Chen G, Wu D, Guo W, Cao Y, Huang D, Wang H (2020). Clinical and immunological features of severe and moderate coronavirus disease 2019. J Clin Invest.

[40] Chen L, Liu HG, Liu W, Liu J, Liu K, Shang J (2020). [Analysis of clinical features of 29 patients with 2019 novel coronavirus pneumonia]. Zhonghua Jie He He Hu Xi Za Zhi.

[41] Braun J, Loyal L, Frentsch M, Wendisch D, Georg P, Kurth F (2020). SARS-CoV-2-reactive T cells in healthy donors and patients with COVID-19. Nature.

[42] Sait A, Angeli C, Doig AJ, Day PJR (2021). Viral involvement in Alzheimer’s disease. ACS Chem Neurosci.

[43] Yin H, Zhang X, Yang P, Zhang X, Peng Y, Li D (2021). RNA m6A methylation orchestrates cancer growth and metastasis via macrophage reprogramming. Nat Commun.

[44] Cheng L, Li H, Zhan H, Liu Y, Li X, Huang Y (2022). Alterations of m6A RNA methylation regulators contribute to autophagy and immune infiltration in primary Sjogren’s syndrome. Front Immunol.

[45] Guo W, Tan F, Huai Q, Wang Z, Shao F, Zhang G (2021). Comprehensive analysis of PD-L1 expression, immune infiltrates, and m6A RNA methylation regulators in esophageal squamous cell carcinoma. Front Immunol.

[46] Zhang B, Wu Q, Li B, Wang D, Wang L, Zhou YL (2020). m(6)A regulator-mediated methylation modification patterns and tumor microenvironment infiltration characterization in gastric cancer. Mol Cancer.

[47] Wyler E, Mosbauer K, Franke V, Diag A, Gottula LT, Arsie R (2021). Transcriptomic profiling of SARS-CoV-2 infected human cell lines identifies HSP90 as target for COVID-19 therapy. iScience.

[48] Qing X, Chen Q, Wang K (2022). m6A regulator-mediated methylation modification patterns and characteristics in COVID-19 patients. Front Public Health.

[49] Dong YM, Sun J, Li YX, Chen Q, Liu QQ, Sun Z (2021). Development and validation of a nomogram for assessing survival in patients With COVID-19 pneumonia. Clin Infect Dis.

[50] Aik W, Scotti JS, Choi H, Gong L, Demetriades M, Schofield CJ (2014). Structure of human RNA N(6)-methyladenine demethylase ALKBH5 provides insights into its mechanisms of nucleic acid recognition and demethylation. Nucleic Acids Res.

[51] Malacrida A, Rivara M, Di Domizio A, Cislaghi G, Miloso M, Zuliani V (2020). 3D proteome-wide scale screening and activity evaluation of a new ALKBH5 inhibitor in U87 glioblastoma cell line. Bioorg Med Chem.

[52] Zannella C, Rinaldi L, Boccia G, Chianese A, Sasso FC, De Caro F (2021). Regulation of m6A methylation as a new therapeutic option against COVID-19. Pharmaceuticals (Basel).

